# Finite element modeling of proximal femur with quantifiable weight-bearing area in standing position

**DOI:** 10.1186/s13018-020-01927-9

**Published:** 2020-09-04

**Authors:** Peng Yang, Tian-Ye Lin, Jing-Li Xu, Hui-Yu Zeng, Da Chen, Bing-Lang Xiong, Feng-Xiang Pang, Zhen-Qiu Chen, Wei He, Qiu-Shi Wei, Qing-Wen Zhang

**Affiliations:** 1 First Clinical Medical College, Guangzhou University of Chinese Medicine, Guangzhou, People’s Republic of China; 2 The Third Affiliated Hospital of Guangzhou University of Chinese Medicine, Joint Orthopedics, Guangzhou, People’s Republic of China; 3 Sun Yat-Sen University Cancer Center, Guangzhou, People’s Republic of China

**Keywords:** Hip joint, Finite element modeling, Weight-bearing area, Image registration

## Abstract

**Background:**

The positional distribution and size of the weight-bearing area of the femoral head in the standing position as well as the direct active surface of joint force can directly affect the result of finite element (FE) stress analysis. However, the division of this area was vague, imprecise, and un-individualized in most studies related to separate FE models of the femur. The purpose of this study was to quantify the positional distribution and size of the weight-bearing area of the femoral head in standing position by a set of simple methods, to realize individualized reconstruction of the proximal femur FE model.

**Methods:**

Five adult volunteers were recruited for an X-ray and CT examination in the same simulated bipedal standing position with a specialized patented device. We extracted these image data, calculated the 2D weight-bearing area on the X-ray image, reconstructed the 3D model of the proximal femur based on CT data, and registered them to realize the 2D weight-bearing area to 3D transformation as the quantified weight-bearing surface. One of the 3D models of the proximal femur was randomly selected for finite element analysis (FEA), and we defined three different loading surfaces and compared their FEA results.

**Results:**

A total of 10 weight-bearing surfaces in 5 volunteers were constructed, and they were mainly distributed on the dome and anterolateral of the femoral head with a crescent shape, in the range of 1218.63–1,871.06 mm^2^. The results of FEA showed that stress magnitude and distribution in proximal femur FE models among three different loading conditions had significant differences, and the loading case with the quantized weight-bearing area was more in accordance with the physical phenomenon of the hip.

**Conclusion:**

This study confirmed an effective FE modeling method of the proximal femur, which can quantify the weight-bearing area to define a more reasonable load surface setting without increasing the actual modeling difficulty.

## Introduction

FE technology plays an important role in digital orthopedic researches. Among them, as the largest weight-bearing joint in the human body, hip joint-related FEA study has always been the research focus [1]. At present, hip joint biomechanical researches are mainly based on a gait-analysis-based model or specific body movement posture model for mechanical analysis. To simplify the calculation process, researchers also use the static hip joint model in a single-legged standing posture as the research object and analogize other motion states by specific linear proportional relations [2].

In our previous research, we established the standing hip model to perform mechanical research and recognized that in the standing position, the contact area as well as the weight-bearing area of the femoral head as the direct active surface of the joint force, its positional distribution and size can directly affect the stress distribution of the femoral head [3]. Studies [4, 5] believed that the weight-bearing area of the femoral head was mainly located on the anterolateral side of the femoral head, and the contact area of the femoral head to acetabular was approximately to be circular. However, we know that the shape of the contact between the acetabulum and the femoral head is elliptical. Furthermore, the circular contact may cause errors in the finite element simulation and is more likely to cause stress concentration in the femoral head, which can affect the authenticity and accuracy of FEA. So, before a biomechanical FEA of the separate femoral head be carried out, it is necessary to define the weight-bearing area scientifically. Genda et al. [6] reported a method for calculating the hip joint contact area in the single-legged standing posture by X-ray film and verified its accuracy, but this method has not been applied to a real case-based FE model. We assume that there is a trade-off of easiness and accuracy between the full hip model and the simplified model.

The purpose of this study is to explore the feasibility of using a set of simple methods to reconstruct the individualized proximal femur FE model with a quantifiable weight-bearing area of the femoral head in standing position, and provide a design idea of quantitative analysis for more accurate FE study.

## Methods

### Patients and study design

**Fig. 1:**
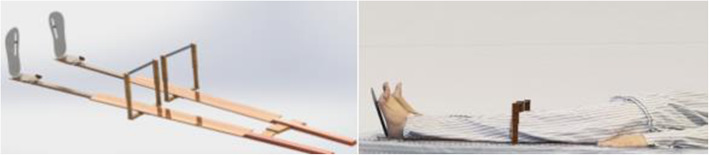
A specialized patented device designed to ensure the same posture fixation while X-ray and CT were performed

Five adult volunteers (2 males, 3 females, as shown in Table 1) without history of hip pain, lower limb disease, or any other systemic diseases were recruited, and subjects knew about the test scheme. X-ray and computerized tomography (CT) were conducted in all volunteers with the same position: simulated bipedal standing in a supine position, bilateral anterior superior iliac spines at the same horizontal line, knees straight, patella up, and heels together with the toes 30° apart. Anteroposterior (AP) view of the pelvis (with a magnification marker): X-ray beam perpendicular to the table, centered at the midpoint between the superior margin of the symphysis pubis and at the midpoint between the anterior superior iliac spines. CT scan (Aquilion 64, Toshiba Medical System Corp., Japan): 0.5-mm slice thickness and 5-mm interval, radiograph ranged from 1 cm above the highest point of the iliac crest to 5 cm below the lesser trochanter. At the above situation, a specialized patented device (National invention patent of China, NO. 201910159244.7, Fig. 1) was designed to ensure the same posture fixation while X-ray and CT were performed.

**Table 1:** Personal data and anatomical parameters of volunteers

Case no.	Age (years)/sex	BMI (kg/m^2^)	CE angle left/right (°)
1	37/male	26.5	35/37
2	38/female	21.3	26/27
3	33/male	22.6	31/32
4	33/female	24.3	33/30
5	32/female	23.1	33/32

### 3D reconstruction of the proximal femur

CT image data were saved in DICOM format and imported into Mimics 16.0 software (Materialise Corp., Belgium) to reconstruct the 3D model of the cortical bone and cancellous bone of the proximal femur.

### Establishment of the 3D femoral head weight-bearing area based on 2D X-ray radiograph

**Fig. 2:**
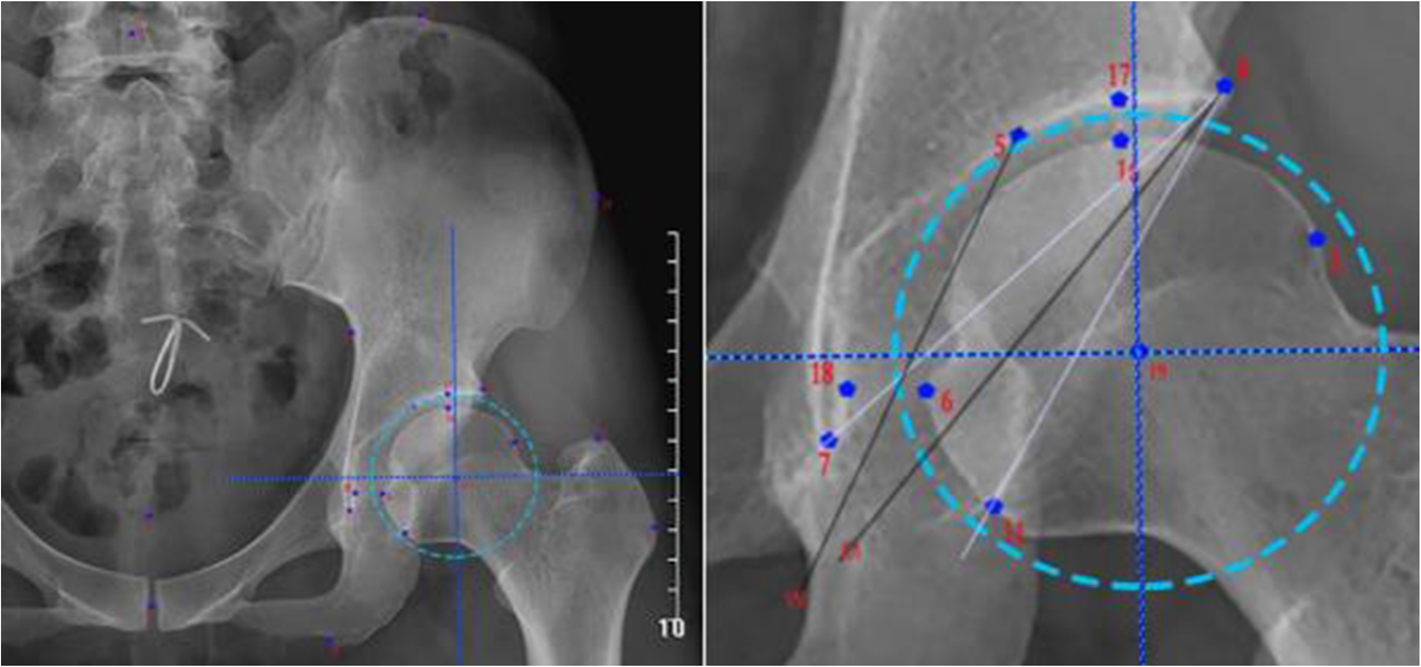
Identification of the anatomical landmarks on the AP radiograph to define the inner and outer edge of the weight-bearing area

The setting of the weight-bearing area was based on the specific anatomical parameters of the X-ray AP view of the pelvis. Firstly, we calculated the magnification of the X-ray film by reference to standard marker (a coin), restored the actual size, and then identified 19 landmark points which included the following (Fig. 2): (1) point in the most lateral of the greater trochanter, (2) point in the top margin of the greater trochanter, (3) point in the most lateral of the femoral head, (4) point in the most lateral of the acetabulum, (5) point in the medial margin of sourcil line, (6) point in the most medial of the femoral head, (7) point in the inferior margin of the teardrop line, (8) midpoint of the line connecting the bilateral teardrop, (9) point in the center of pubic symphysis, (10) point in the inferior margin of the ischium, (11) point in the intersection between the posterior acetabular margin and inferior margin of the femoral head, (12) point in the most lateral of the ilium, (13) point in the inferior margin of the ilium, (14) point in the center of the fifth lumber vertebrae, (15) point in the inner margin of the ilium nearest to point 12, (16) point in the top margin of the femoral head, (17) point in the acetabular contour relative to point 16, (18) point in the lateral margin of teardrop relative to point 6, and (19) center of the femoral head. The calculation of anatomic parameters was referred to Genda et al., which based on the above landmark points: (a) lateral margin of the weight-bearing area (line 4–M): bisecting the angle 7–4–11 and (b) medial margin of the weight-bearing area (line 5-W): connecting point 5 and W (midpoint between points 6 and 18).

### Image registration of the proximal femur 3D reconstruction model and the 2D image of X-ray

**Fig. 3:**
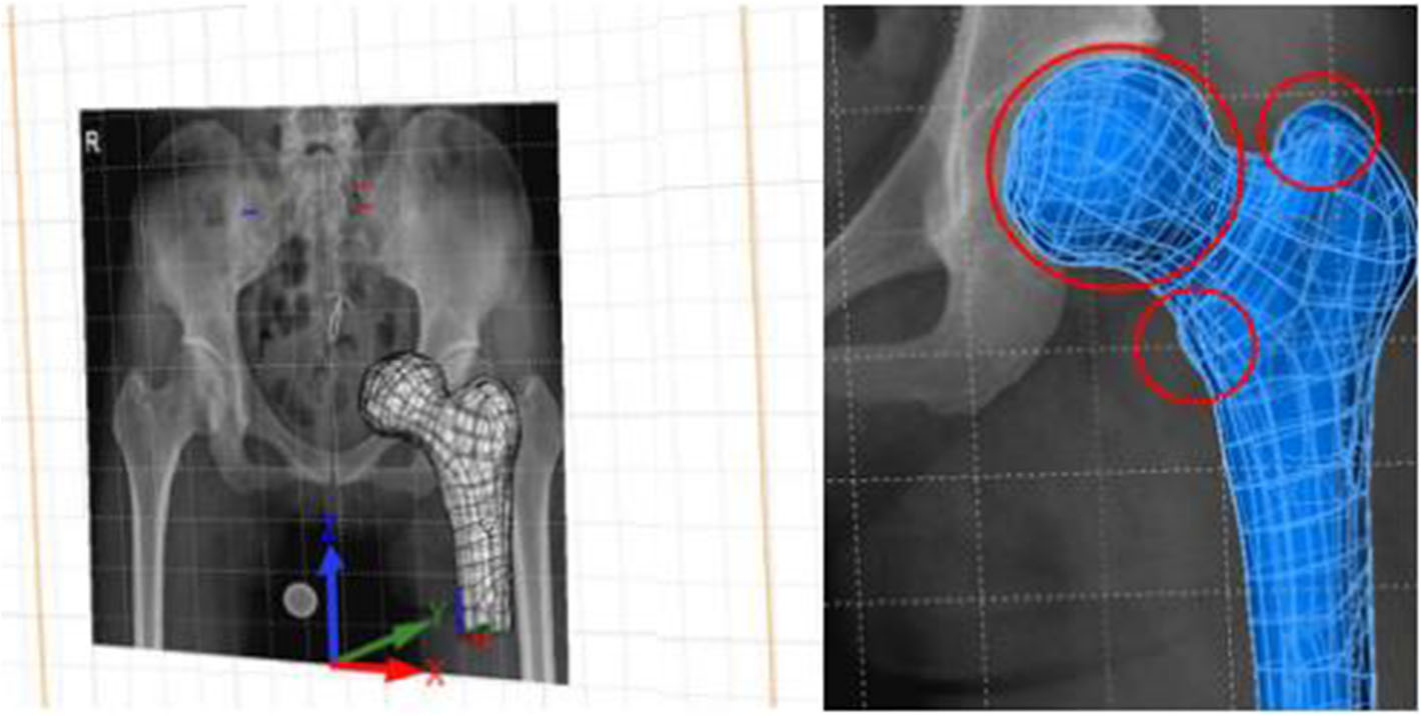
Registration of the proximal femur 3D model and X-ray image based on the greater trochanter, lesser trochanter, and femur contour

A datum plane parallel to the coronal plane of CT was established in Solidworks 2014 software (Dassault Systemes S.A., France) and extracted actual size 2D image of X-ray AP view of the pelvis on this plane. View adjustment of 3D reconstructed graphic of the proximal femur was taken based on X-ray 2D image as reference background to realize the registration of the 3D reconstructed graphic with 2D X-ray image in eliminate edge coloring mode by taking the specific anatomic markers of the lesser trochanter and greater trochanter, femur contour as the registration points (Fig. 3). After satisfactory image registration, a datum plane α parallel to the perspective plane of the window was created.

### Calculation of the 3D weight-bearing area

**Fig. 4:**
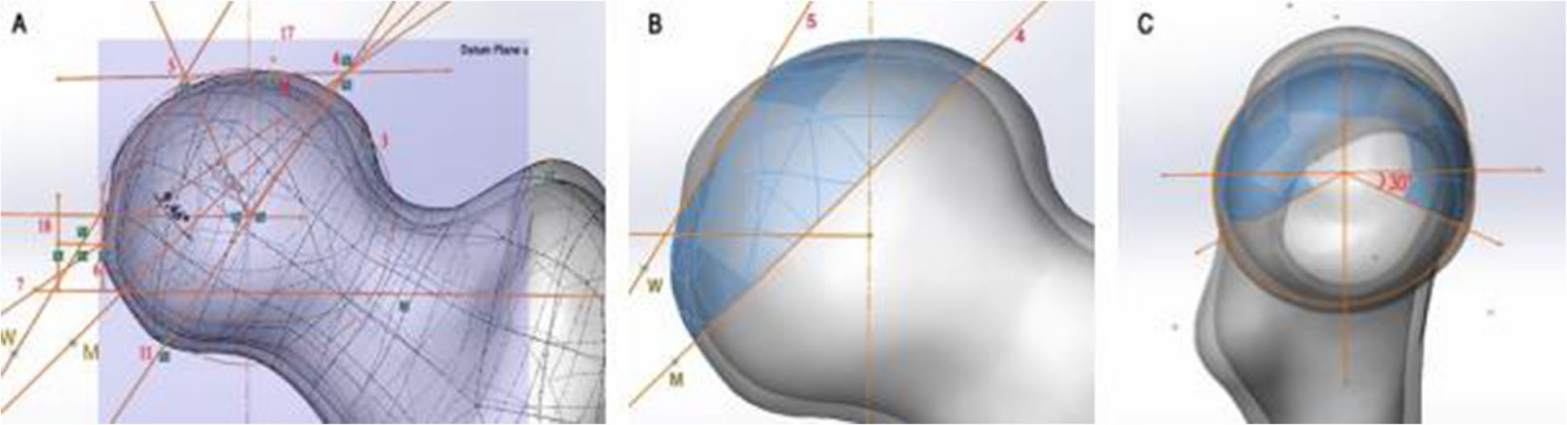
Calculation of the 3D weight-bearing area. **a** Transformation of the landmarks from the X-ray image to the datum plane α. **b**, **c** Definition of the limit line to trim out the shape of the weight-bearing surface

Based on the registered image, the 2D landmark points on the X-ray AP view of the pelvis were transformed into the 3D points on the coordinate system of datum plane α by vertical projecting, and line 4–M and line 5-W were recalculated on datum plane α. Then, line 4–M and line 5-W trimmed surface α generating over the surface of the femoral head with Offset Surface command (0 mm) to become surface β. Referring to Genda et al. [6], we defined 30° below the horizontal plane through the center of the femoral head as the inferior limit of the weight-bearing area and thus trimmed surface β along the direction of joint reaction force to generate surface γ, namely the 3D weight-bearing area (Fig. 4). To facilitate post-processing of FEA, surface γ was filleted in 1 mm. Through the above steps, a total of 10 weight-bearing surfaces and 10 proximal femur 3D models in 5 volunteers were constructed.

### Finite element analysis

#### Mesh generation

**Fig. 5:**
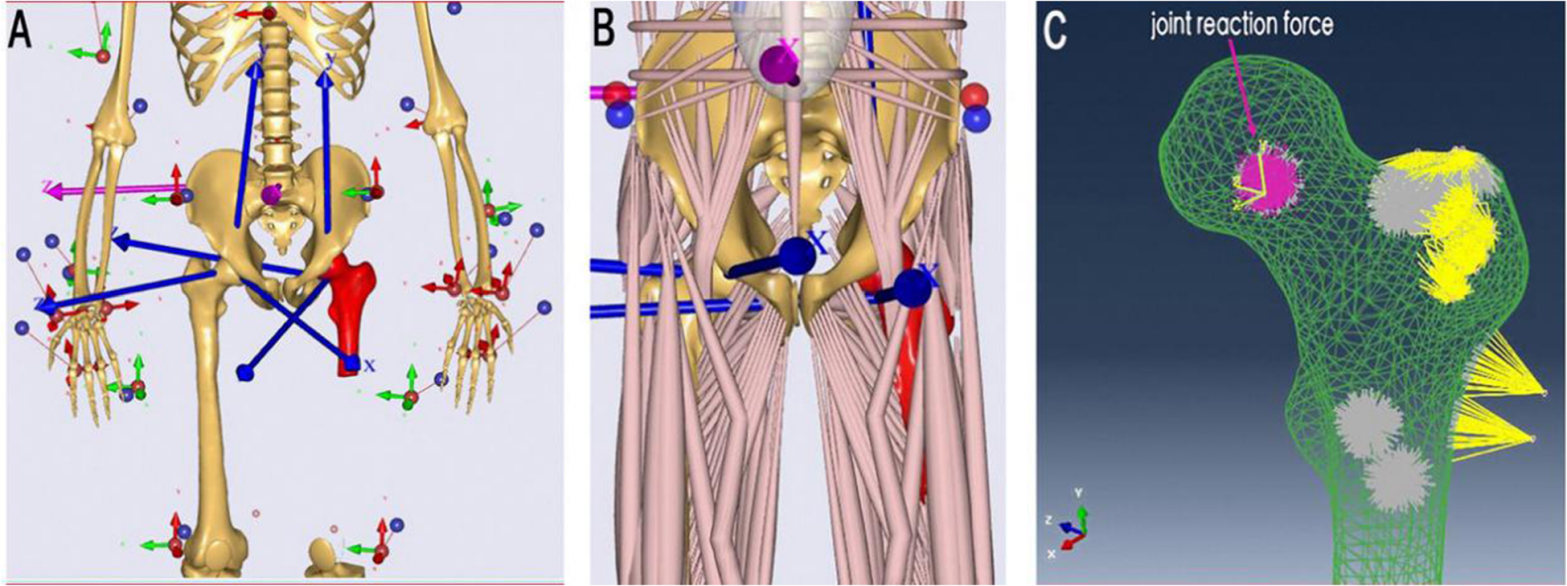
Muscle and joint reaction force. **a**, **b** Musculoskeletal multibody modeling framework (before and after inverse dynamics loading) matched with the proximal femur model. **c** Muscle and joint reaction force was applied to the FE model

One of the above proximal femur models was randomly selected and imported into Abaqus 6.14 software (Dassault Systemes S.A., France) to generate isotropic C3D10 tetrahedral elements with a mesh size of 2 mm. The total number of elements in the cortical bone mesh was 109,684 (210,154 nodes); the cancellous bone mesh was 69,566 (120,336 nodes). Material properties: The simplified model was defined by bi-material properties and material properties used for each component which was referred to literature [7]. Boundary condition: A musculoskeletal multibody modeling framework in standing position was constructed by AnyBody Modeling System version 6.1 (AnyBody Technology, Denmark) and matched with the proximal femur 3D reconstruction model; then, inverse dynamic loading was performed, the muscle and joint reaction force data (magnitude and direction) during standing was obtained, and the data was exported to the FE model for the boundary condition setting, as shown in Fig. 5. Constraints were applied to the distal end of the model, and all six degrees of freedom were constrained to zero.

#### Joint reaction force

**Fig. 6:**
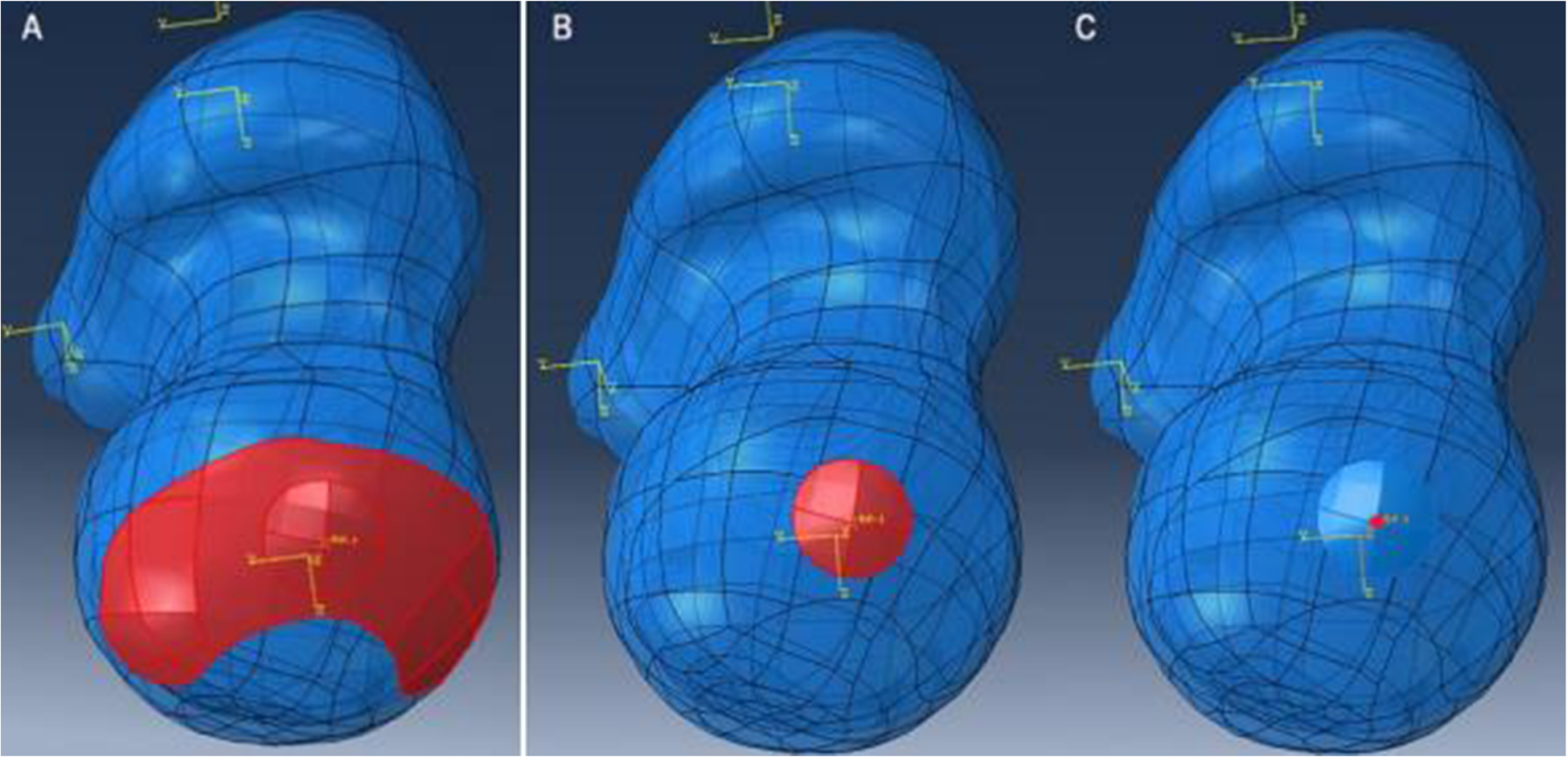
Models with different loading conditions

To observe the effect of different joint reaction force areas on the internal stress distribution of the femoral head, three different loading conditions were simulated, as shown in Fig. 6. In the first loading case A, joint reaction force was applied to the weight-bearing area (surface γ) being established above. Two common joint reaction force loading methods in literature were designed as loading case B (the circular region with diameter 2 cm at the top of the femoral head) and loading case C (apical point of the femoral head), respectively. The joint reaction force simulating a single-legged stance in three cases was loaded onto each corresponding coupling surface of the joint reaction force area. Meanwhile, a normal hemipelvis and entire hip joint (hemipelvis-hip) model which has been established in our previous study [8] was introduced to compare with the models described above, and the same load as the ground reaction force was applied to the hemipelvis-hip model.

#### Defining path

To better analyze the internal stress change in the femoral head, a path α was defined inside the model from the vertex (node serial number 2,716) to the head-neck junction (node serial number 62,626) on the *Y*-axis sagittal section (position serial number -2.85069). Twelve mesh elements were picked up on path α and recorded the stress values in principal stress orientation.

## Results

### Weight-bearing area distribution and size

**Fig. 7:**
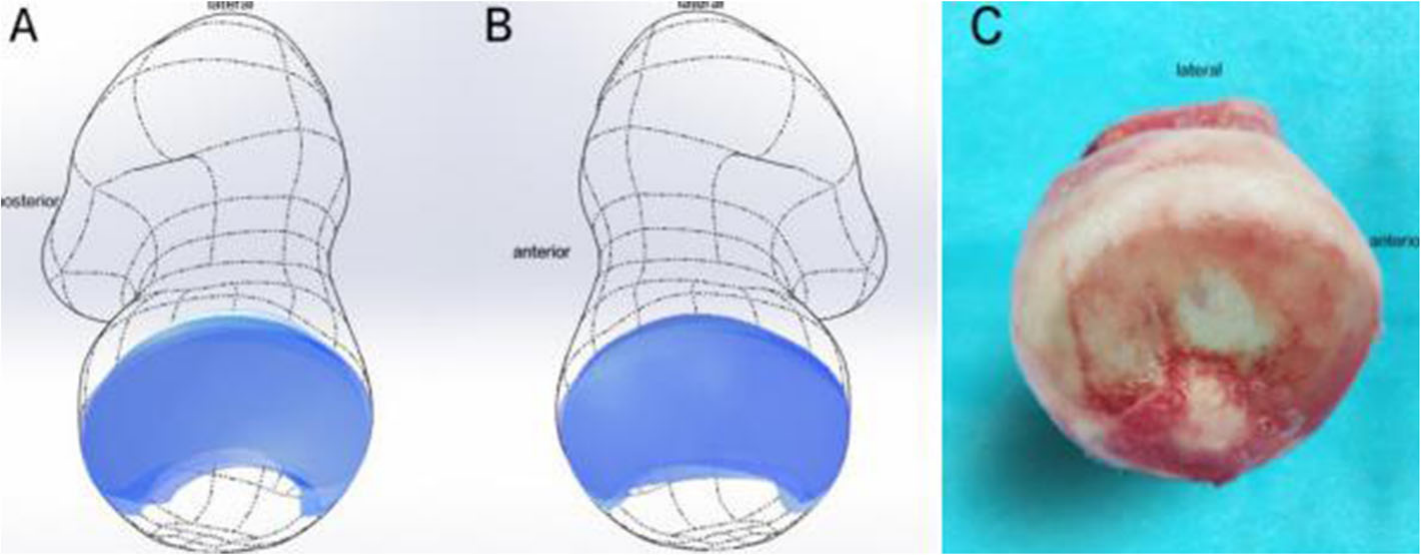
Distribution of the weight-bearing area on the femoral head surface. **a** Left side in 5 volunteers. **b** Right side in 5 volunteers. **c** A femoral head of arthritis patient removed in joint replacement surgery with obvious cartilage wear in the weight-bearing area

In this study, a total of 10 weight-bearing surfaces in 5 volunteers were constructed through the individualized process. The surfaces were mainly distributed on the dome and anterolateral of the femoral head with a crescent shape, as shown in Fig. 7. Quantifying the size: the maximum area was 1871.06 mm^2^, and the minimum area was 1218.63 mm^2^. The results of literature comparison are shown in Table 2.

**Table 2:** The size and distribution of the weight-bearing area with literature contrast

Literature	Size	Distribution
Wang et al. [9]	1470 mm^2^ (acetabular)	Dome of acetabular
Greenwald and Haynes [10]	2002 mm^2^ (containing cartilage)	Dome and anterolateral of the femoral head
Brown and Shaw [11]	1700 mm^2^	Dome and anterolateral of the femoral head
Our study	1218.63–1871.06 mm^2^	Dome and anterolateral of the femoral head

### Stress magnitude and distribution

**Fig. 8:**
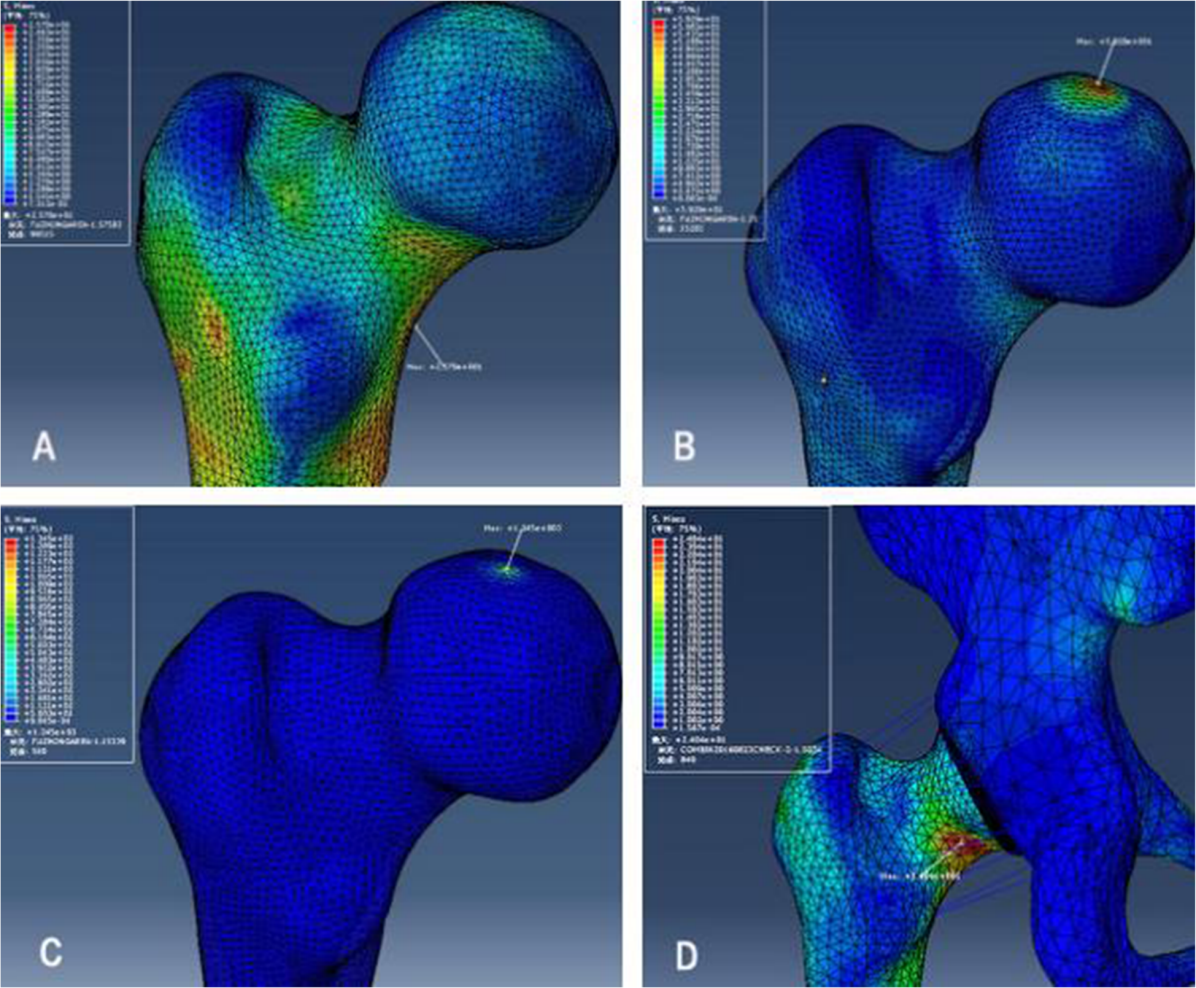
The distribution of maximum Von Mises stress in the femoral cortical bone. **a**–**c** Models in loading cases A, B, and C. **d** The hemipelvis-hip model

**Fig. 9:**
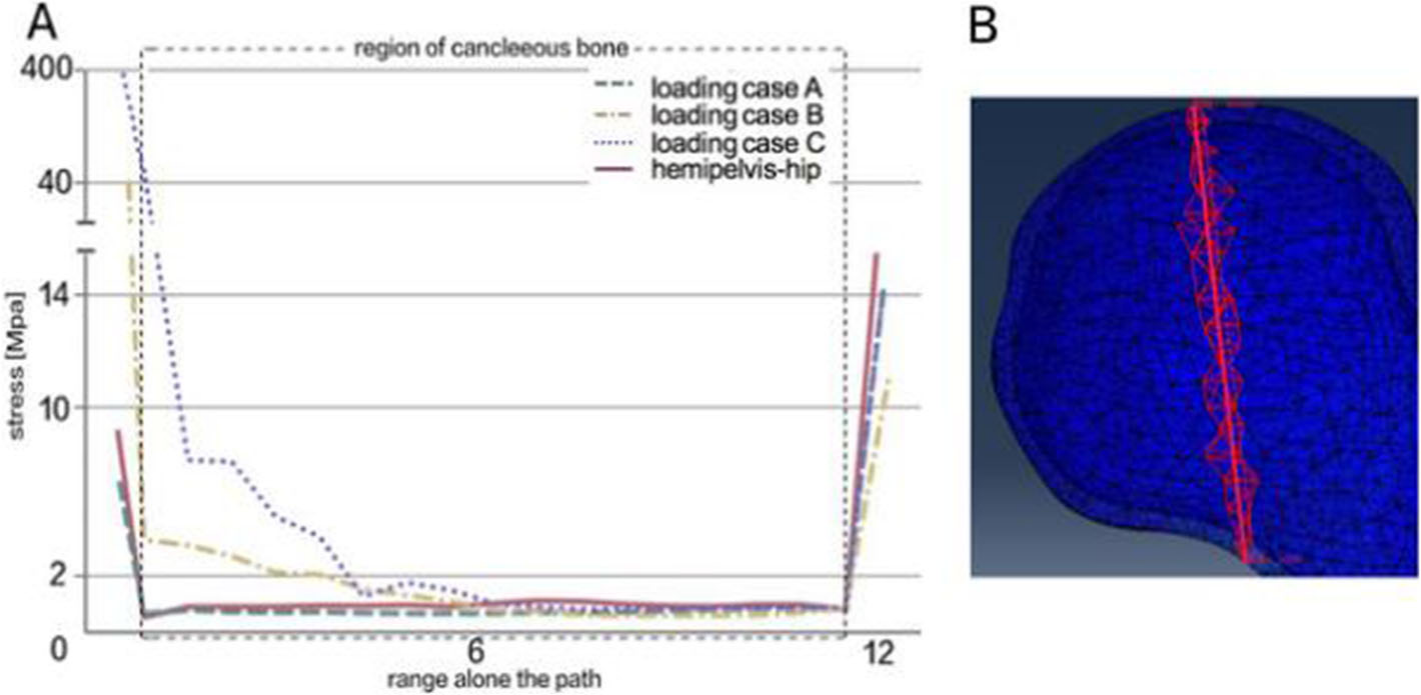
Comparison of principle stress changes in different loading conditions on path α. **a** Stress magnitude and distribution in proximal femur FE models among three different loading conditions had significant differences (*p* < 0.05); the loading case A (quantized weight-bearing area) was more in accordance with the hemipelvis-hip model. **b** Definition of path α

**Fig. 10:**
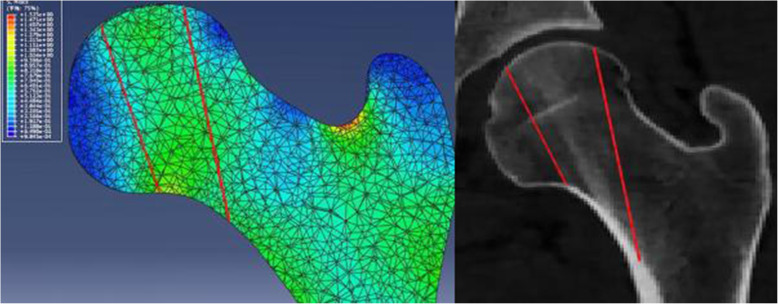
Stress cloud chart showing a stress concentration area appeared in the femoral head that was coherent with the stress bone trabecula located in the femoral head

The results of FEA showed that stress magnitude and distribution of proximal femur FE models in A, B, and C three different loading conditions had significant differences. In loading case A with the quantized weight-bearing area, the maximum stress (25.7 MPa) of the cortical bone located in the region of femoral neck-body junction, which was similar to that of the hemipelvis-hip model (maximum stress was 24.04 MPa), and different to that of loading cases B and C, whose stress concentration appeared at the top region of the femoral head (the maximum stress was 59.29 MPa and 13.45 MPa, respectively) where the joint reaction force applied to, as shown in Fig. 8. Analysis of internal stress distribution of the femoral head: The stress values of 12 elements on path α in different loading conditions were extracted to plot the graph (Fig. 9), and we discovered that stress patterns in loading case A and the hemipelvis-hip model shared strong similarities, which was significantly different from loading cases B and C. In loading case A, the internal stress concentration region loading principal stress reflected mechanical transfer path, whose region and sharp were consistent with the physiological distribution of compression trabeculae (Fig. 10), while this feature was not evident in either loading case B or C. Hence, the FEA results of loading case A with the quantized weight-bearing area are more in accordance with the physical phenomenon of the hip.

## Discussion

Abnormal biomechanical process is an important factor related to the development and progression of hip disease, and FEA technology can be well used to study those mechanical characteristics and reveal the changing rules to guide treatment. Taking osteonecrosis of the femoral head (ONFH) as an example, collapse is the most critical process in the four main pathological changes [12] (necrosis, repair, collapse, and osteoarthritis); previous studies have shown that the occurrence of collapse is the result of the interaction of biological and biomechanical factors [13]. In our prophase research, we have established the FE model of the hemipelvis and entire hip joint containing necrosis [3] and expounded that one of the mechanical mechanisms of collapse is the stress shielding effect leading to the uneven distribution of stress transmission in the femoral head and the excessive concentration of stress on the surface (underlying subchondral bone) of the femoral head. And on this basis, we optimized focus debridement being used in fibular allograft with impaction bone grafting to treat ONFH. However, the conduction of stress in the femoral head is actually affected by many factors, chief among them is the weight-bearing area of the femoral head as the direct active surface of the joint force in standing posture [10, 14]. In the research on FEA, the distribution and size of the weight-bearing area can be well reflected by establishing a pelvis and entire hip joint model [15–18]. However, in the FE modeling approach of the pelvis and entire hip joint, the numerical simulation of cartilage and complex algorithm defining for joint contact relationship need to be considered. These processes are tedious and time-consuming, which may increase the risk of subjective bias and diminish the validity of the result, especially in the large sample test. For certain cases, researchers prefer constructing the separate FE model of the femur (or proximal femur) to perform related mechanical analysis to improve the efficiency of FEA, which can be considered as a feasible alternative to simplify the FE model of the hip [19–22].

Different from the FE model of the pelvis and entire hip joint, the region of joint reaction force as well as the weight-bearing area in such separate model with standing position needs to be defined before stress loading. Most of the related literature present to design the weight-bearing area as an ambiguous elliptical region [23, 24], or load the joint force by simplifying it into a point [19, 25]. This can unify load condition and quickly realize the mechanical analysis. But in reality, the weight-bearing area is not nearly as elliptical or point. Kummer [26] suggested that the weight-bearing area should be the overlap between the upper hemisphere of the femoral head and the acetabulum, known as the “spherical binangle.” Daniel et al. [27] calculated by formula that the weight-bearing area should be in the area where the top of the femoral head overlaps with the acetabular cartilage surface, and can be affected by the shape of the acetabular cartilage surface. Greenwald and Haynes [12] through the experiment of 51 cadaveric specimens obtained the fan-shaped weight-bearing area in standing position distributing at the dome of the femoral head. Bachtar et al. [28] also showed the crescent-shaped weight-bearing area at the superior and anterosuperior parts of the femoral head by the virtual FE simulation of the hip with the Gregory patch smoothing algorithm for contact elements. As shown in this research, we quantified the size and positional distribution of 10 weight-bearing areas of the femoral head in standing position through an individualized process, and results of literature comparison confirm the reliability of the study. The results of FEA demonstrate that the difference of the weight-bearing area leads to a significant difference in stress distribution; the setting of the FE model with the quantized weight-bearing area is more in accordance with the physiological situation of the hip.

The calculation method of the weight-bearing area of the femoral head in standing position mainly refers to the theoretical formula proposed by Genda et al. [6], and the 3D reconstruction is realized by the principle of projection transformation and image registration. Ishimaru et al. [29] reported simulating X-ray by projection transformation to create a virtual radiographic image of a 3D knee joint model and registering it with real X-ray film to realize visual reconstruction of polymer polyethylene patellar component and radiographically determine its external contour. Image registration is vital to the accurate construction of the weight-bearing area in our modeling process, and obtaining X-ray, CT data of one volunteer in the same body posture is the premise of accurate registration. So, we designed a patented device to ensure the same posture fixation while X-ray and CT were performed. The results of FEA show that the FE model of the proximal femur with the quantized weight-bearing area can successfully carry out force analysis and is more in accordance with the physical phenomenon of the hip-femur. Compared with the traditional FE modeling method, the proposed method is more accurate and reasonable for the load surface setting without increasing the actual modeling difficulty. This research significance lies in that it established a quantifiable model basis for further exploring the biomechanical effects of changes in the weight-bearing area on the occurrence and development of femoral head-related diseases. Simultaneously, it also provided a quantitative analysis design idea for more accurate FE research.

One of the limitations of this study is that the shape and direction of the acetabulum cannot be accurately judged through the 2D film alone, thus ignoring its influence on the distribution of the weight-bearing area, which may lead to inaccuracy on the sagittal plane. Furthermore, the process of image registration mainly depended on manual work, so the accuracy of registration could be affected by subjective factors. Therefore, a true 3D FE model should be performed based on CT and MRI data including the entire hip joint and pelvis to assure the reliability of the simulation. This study explored the feasibility of a more realistic and quantifiable simplified modeling method, which can particularly be applied for rapid and individual modeling of large sample. However, further works are required to consider the correlation and translation between X-ray and CT images in the shape and direction of acetabular, which will be based on sufficient clinical research data. And automatic image registration will be considered to reduce subjective bias by means of coordinate point registration.

## Conclusion

This study confirmed an effective FE modeling method of the proximal femur, which can quantify the weight-bearing area to define a more reasonable load surface setting without increasing the actual modeling difficulty, and those researches which involve the FEA of the femoral side could benefit.

## Data Availability

All data and materials are contained within the manuscript.

## Abbreviations

FE: Finite element
FEA: Finite element analysis
CT: Computerized tomography
AP: Anteroposterior
